# Use of pharmacogenomics in elderly patients treated for cardiovascular diseases

**DOI:** 10.3325/cmj.2020.61.147

**Published:** 2020-04

**Authors:** Nada Božina, Majda Vrkić Kirhmajer, Livija Šimičević, Lana Ganoci, Nikica Mirošević Skvrce, Iva Klarica Domjanović, Iveta Merćep

**Affiliations:** 1 Department of Pharmacology, University of Zagreb School of Medicine, Division of Pharmacogenomics and Therapy Individualization, Department of Laboratory Diagnostics, University Hospital Centre Zagreb, Zagreb, Croatia; 2 Department of Cardiovascular Diseases, University Hospital Centre Zagreb, University of Zagreb School of Medicine, Zagreb, Croatia; 3 Division of Pharmacogenomics and Therapy Individualization, Department of Laboratory Diagnostics, University Hospital Centre Zagreb, Zagreb, Croatia; 4 Croatian Agency for Medicinal Products and Medical Devices, Zagreb, Croatia; 5 Department of Internal Medicine, University Hospital Centre Zagreb, University of Zagreb School of Medicine, Zagreb, Croatia

## Abstract

Older people are increasingly susceptible to adverse drug reactions (ADRs) or therapeutic failure. This could be mediated by considerable polypharmacy, which increases the possibility of drug-drug and drug-gene interactions. Precision medicine, based on individual genetic variations, enables the screening of patients at risk for ADRs and the implementation of personalized treatment regimens. It combines genetic and genomic data with environmental and clinical factors in order to tailor prevention and disease-management strategies, including pharmacotherapy. The identification of genetic factors that influence drug absorption, distribution, metabolism, excretion, and action at the drug target level allows individualized therapy. Positive pharmacogenomic findings have been reported for the majority of cardiovascular drugs (CVD), suggesting that pre-emptive testing can improve efficacy and minimize the toxicity risk. Gene variants related to drug metabolism and transport variability or pharmacodynamics of major CVD have been translated into dosing recommendations. Pharmacogenetics consortia have issued guidelines for oral anticoagulants, antiplatelet agents, statins, and some beta-blockers. Since the majority of pharmacogenetics recommendations are based on the assessment of single drug-gene interactions, it is imperative to develop tools for the prediction of multiple drug-drug-gene interactions, which are common in the elderly with comorbidity. The availability of genomic testing has grown, but its clinical application is still insufficient.

Variability in the response to drug therapy is a widespread issue. It may be affected by various factors, including, but not limited to age, sex, renal and hepatic function, as well as drug-drug and drug-food interactions. An important role is also played by genetics (1); genetic predisposition accounts for approximately 20%-40% of interindividual variability in drug response (2), but in the case of some therapies, eg, metoprolol and torsemide, twin studies revealed that genetic contribution to pharmacokinetic (PK) variability is up to 90% (3). Potential effects of genetic polymorphisms are numerous and include prolonged and enhanced pharmacological effect, drug toxicity and side effects, the lack of efficacy in the use of recommended doses and need for higher doses, activation of alternative and harmful biochemical pathways, as well as drug interactions.

Genetic profiling has been first implemented in the area of pharmacogenetics/pharmacogenomics. While pharmacogenetics investigates a specific DNA polymorphism or coding variant, pharmacogenomics investigates the role of various genome components in the response to a drug. Personalized medicine (also termed precision medicine) refers to an approach that uses a patient-unique profile. It combines genetic and genomic data with environmental and clinical factors to assess individual risks and tailor prevention and disease-management strategies, including pharmacotherapy.

The major challenge in pharmacogenomics is translating the results of genetic testing into treatment recommendations. In recent years, genotype-based guidelines have provided strong evidence linking genetic variants to the variability of drug efficacy or risk for the development of adverse reactions (ADRs) (4). This is of enormous importance for the elderly patients since the risk for ADRs increases with age, comorbidity, and the number of concomitant medicinal therapies (5). Among others, the Clinical Pharmacogenetics Implementation Consortium (CPIC) has issued very helpful guidelines for dosing of different medicines according to related pharmacogenes. The pharmacogenetic data may improve our ability to select the most appropriate medication, individualize the dose and dosing schedule, thus yielding significant health and economic benefits for patients and society (6).

In this review, we would like to present some recommendations and guidelines considering pharmacogenomics for the treatment of the elderly with comorbidity and polypharmacy, giving specific examples of pharmacogenomics of cardiovascular drugs applied in the elderly population. We also summarized relevant published data from our previous investigations and scientific literature.

## Adverse drug reactions in the elderly

Adverse drug reactions (ADRs) are a significant cause of morbidity, mortality, and health care costs and are responsible for nearly 30% of hospital admissions of elderly patients (7,8). Furthermore, ADRs in the elderly population are expected to be more severe and underreported, with a substantially high mortality rate. More than 80% of ADRs as cause of admission or registered in hospital are related to the applied dose, which makes them predictable and avoidable (9,10). A meta-analysis in the US found that ADRs contributed to 100 000 deaths per year (11), while a Swedish study reported that they contributed to 3.1% deaths per year in the general population (12). The overall mortality of hospitalized patients linked to an ADR ranges from 0.14% to 4.7%, with fatal outcomes more likely occurring in patients older than 55 and the greatest risk in patients older than 75 (11,13). The most important risk factors for ADR-related hospitalizations were older age, comorbidities with polypharmacy, and possibly unsuitable medicines. ADR-related hospital admissions are mostly attributable to antiplatelets, anticoagulants, diuretics, NSAIDs, and antidiabetic drugs (14). ADR-related hospitalization in older patients can be prevented by the development of intervention strategies and prediction tools (15). By increasing drug efficacy and drug safety and decreasing ADRs, the potential for cost savings is enormous (11).

## Pharmacogenomics of ADRs

Pharmacogenetic variability could influence drug response at the PK and pharmacodynamics (PD) level. When applying the equal dose of a drug to two unrelated persons of equal weight, plasma drug level differences can be more than 1000-fold (2,16). Pharmacokinetics investigates the transport of administered drugs, including their absorption, distribution, metabolism, and excretion (processes coded by ADME genes). ADME genes are highly variable (17,18), and this variability significantly accounts for interindividual inconsistency in medication efficacy and toxicity (2). The majority of pharmacogenetics studies reported on genetic differences in drug-metabolizing enzymes of phase I (predominantly P450 CYPs) and phase II, as well as drug transporters. The effects of pharmacogenetics on PD refer to genetic variations of the drug targets (ie, receptors), with the consequence of a decreased therapeutic efficacy. The identification of genetic factors that underlie these differences might optimize drug efficiency and improve the safety profile, thus enabling individualized therapy (19).

## Polypharmacy

Older people often suffer from comorbidities, resulting in a high use of polypharmacy. Multiple studies confirmed polypharmacy to be a considerable risk for drug-disease, drug-drug, and drug-gene interactions (5,20). The consequences of polypharmacy include ADRs, admission to hospitals, fatal outcomes, and other health adverse outcomes. Elderly patients with altered drug metabolism or PD can be identified by pharmacogenetic testing. Published data has confirmed the potential of precision medicine, especially in older patients with polypharmacy and with a history of emergency care admission (21). However, predicting the deleterious impact of polypharmacy still requires comprehensive research that should take into consideration structured drug-drug and drug-drug-gene interactions, which are common in this population. Recently, enzymes and transporters included in drug metabolism have been analyzed for the 100 top-selling drugs that already had pharmacogenetic data in their summaries of product characteristics (22). Such an approach could improve the identification of combinatorial pharmacogenomic associations.

Many drugs prescribed to older adults are metabolized by multiple cytochrome P450 (CYP) enzymes, which participate in the biotransformation of 70%-90% of overall approved drugs. The most common CYPs responsible for drug biotransformation are CYP2C9, CYP2C19, CYP2D6, CYP3A4, and CYP3A5 (23). Genotypes can help us predict patients’ enzymatic activity, ranging from poor to ultrarapid (4). Besides considerable interindividual variability, there are also significant interpopulation and interracial differences in CYP polymorphisms frequency (24).

An increasing number of applied drugs increases the risk for multiple drug-drug and drug-gene interactions, with probable ADRs. Liu et al described how pharmacogenomics can be used for drug risk assessment in patients on polypharmacy (25).

## Pharmacogenomics of cardiovascular drugs

The significance of pharmacogenomics (PGx) was confirmed for 72% of cardiovascular drugs, which further resulted in clinically actionable PGx information (26). Table 1 shows gene-cardiovascular drug pairs for which PGx societies, mainly the CPIC and**Dutch Pharmacogenetics Working Group (DPWG), issued recommendations.

**Table 1:** The summary of recommendations from guidelines for cardiovascular drug dosing according to genotypes, issued by DPWG and CPIC*^†^

Drug	Gene/allele	Genotype	Clinical effects	Recommendation	Guidelines
Acenocoumarol	*VKORC1* *-1639 G>A*	AA	The genetic variation increases the sensitivity to acenocoumarol.	Patients with the *VKORC1-1639 AA* genotype are recommended to be given 50% of the standard initial dose of acenocoumarol and undergo more frequent monitoring of INR.	DPWG Guideline for acenocoumarol and VKORC1 (77,78)
Atorvastatin	*SLCO1B1* *421 t > C*	CC TC	The genetic polymorphism may lead to reduced atorvastatin transport to the liver. This may increase atorvastatin plasma concentrations and therefore the risk of myopathy.	An alternative drug for patients with the *SLCO1B1 521 CC* or *TC* genotype and with additional significant risk factors for statin-induced myopathy.	DPWG Guideline for atorvastatin and SLCO1B1 (77,78)
Clopidogrel	*CYP2C19* *2, *3, *17	PM (*2/*2, *2/*3, *3/*3)	Significantly reduced platelet inhibition; increased residual platelet aggregation; increased risk for adverse cardiovascular events.	Alternative antiplatelet therapy (eg, prasugrel, ticagrelor) if there is no contraindication.	CPIC Guideline for clopidogrel and CYP2C19 (46)
		IM (*1/*2, *1/*3, *2/*17)	Reduced platelet inhibition; increased residual platelet aggregation; increased risk for adverse cardiovascular events.	Alternative antiplatelet therapy (eg, prasugrel, ticagrelor) if there is no contraindication.	
		UM (*1/*17,*17/*17)	Increased platelet inhibition; decreased residual platelet aggregation. The genetic variation may be associated with increased risk of bleeding.	Clopidogrel – label recommended dosage and administration.	
Flecainide	*CYP2D6*	PM	The genetic variation reduces conversion of flecainide to inactive metabolites. This increases the risk of side effects.	Reduce the dose to 50% of the standard dose and record an ECG and monitor the plasma concentration.	DPWG Guideline for flecainide and CYP2D6 (77,78)
		IM		Reduce the dose to 75% of the standard dose for CYP2D6 intermediate metabolizer (IM) patients with indications other than the diagnosis of Brugada syndrome and record an ECG and monitor the plasma concentration.	
		UM	The genetic variation increases conversion of flecainide to inactive metabolites. A higher dose is possibly required as a result.	There are no data about the pharmacokinetics and/or the effects of flecainide in UM.	
Metoprolol	*CYP2D6*	PM	The gene variation reduces the conversion of metoprolol to inactive metabolites. However, the clinical consequences are limited mainly to the occurrence of asymptomatic bradycardia.	If a gradual reduction in heart rate is desired, or in the event of symptomatic bradycardia, prescribe no more than 25% of the standard dose, increase the dose in smaller steps.	DPWG Guideline for metoprolol and CYP2D6 (77,78)
		IM		If a gradual reduction in heart rate is desired, or in the event of symptomatic bradycardia, prescribe no more than 50% of the standard dose, increase the dose in smaller steps.	
		UM	The gene variation increases the conversion of metoprolol to inactive metabolites. This can increase the dose requirement. However, with a target dose of 200 mg/d, there was no effect on the blood pressure and hardly any effect on the reduction of the heart rate.	Use the maximum dose for the relevant indication as a target dose, and if the effectiveness is still insufficient: increase the dose based on effectiveness and side effects to 2.5 times the standard dose or select an alternative drug.	
Phenprocumon	*VKORC1* *-1639 G>A*	AA	The genetic variation increases the sensitivity to phenprocoumon.	Patients with the VKORC1*-1639 (rs9923231) AA* genotype are recommended to be given 50% of the standard initial dose of phenprocoumon and more frequent monitoring of INR.	DPWG Guideline for phenprocoumon and VKORC1 (77,78)
Propafenone	*CYP2D6*	PM	Genetic variation increases the sum of the plasma concentrations of propafenone and the active metabolite 5-hydroxypropafenone. This increases the risk of side effects.	Reduce the dose to 30% of the standard dose, perform an ECG and monitor plasma concentrations.	DPWG Guideline for prophafenone and CYP2D6 (77,78)
		IM		Monitor plasma concentrations and perform an ECG or select an alternative antiarrhythmic drug.	
		UM	Genetic variation decreases the sum of the plasma concentrations of propafenone and the active metabolite 5-hydroxypropafenone. This increases the risk of reduced or no efficacy.	Monitor plasma concentrations and perform an ECG or select an alternative antiarrhythmic drug.	
Simvastatin	*SLCO1B1* *421 t > C*	CC	High myopathy risk	Prescribe a lower dose or consider an alternative statin (eg, pravastatin or rosuvastatin); consider routine CK surveillance.	CPIC Guideline for simvastatin and SLCO1B1 (63,65)
		TC	Intermediate myopathy risk	Prescribe a lower dose or consider an alternative statin (eg, pravastatin or rosuvastatin); consider routine CK surveillance.	
Warfarin	*CYP2C9* *2,*3			The genotype-specific initial dose and maintenance dose can be calculated using an algorithm, at *www.warfarinDosing.org*	CPIC Guideline for warfarin and CYP2C9,CYP4F2, VKORC1 (27,28)
	*VKORC1* *-1639 G>A*				
	*CYP4F2* (rs2108622)				

*INR – international normalized ratio; IM – intermediate metabolizer; PM – poor metabolizer; UM – ultrarapid metabolizer; CPIC – The Clinical Pharmacogenetics Implementation Consortium (*https://cpicpgx.org/guidelines/*); DPWG – The Dutch Pharmacogenetics Working Group (*https://www.knmp.nl/downloads/pharmacogenetic-recommendations-february-2020.pdf*).

†Adapted according to PharmGKB – The Pharmacogenomics Knowledgebase, (*https://www.pharmgkb.org/guidelineAnnotations*).

### Vitamin K antagonists (VKAs)

The pharmacogenetics of VKAs, coumarin-type anticoagulants, is based on genetic polymorphisms related to overdosing risk and to resistance. The variability of warfarin exposure and risk for bleeding is mostly explained by the gene variants that code proteins involved in the PK and PD of VKAs: cytochrome P450 isoform 2C9 (CYP2C9) and vitamin K epoxide reductase subunit 1 (VKORC1).

The Food and Drug Administration, followed by the European Medicines Agency, recognized the utility of the *CYP2C9* and *VKORC1* genotypes, together with non-genetic factors. Today, the algorithm applications (eg, *www.WarfarinDosing.org*) take into account the mentioned genes variants to define the recommended initial VKAs dose. Data on these genes are noted in the warfarin summary of product characteristics. Subsequently, the CPIC and DPWG issued warfarin dosing guidelines (27,28).

Our data obtained for patients with ischemic stroke showed that the introduction of genotype-guided administration in early stages of warfarin treatment shortened the required stabilization period and enhanced anticoagulant effectiveness, with improved clinical outcomes, crucial in clinical practice (29,30). Furthermore, the economic evaluation of such an approach indicated that PGx-guided warfarin therapy was a cost-effective treatment decision for the management of older patients with atrial fibrillation (AF) who developed ischemic stroke (31).

### Direct oral anticoagulants

In recent years, direct oral anticoagulants (DOACs) have been increasingly prescribed owing to their favorable PK and PD, hence they do not require routine coagulation monitoring. Nevertheless, the cases of inter-individual variability in plasma DOACs levels and unexpected bleeding complications were documented, prompting PGx studies of the most frequently used DOACs (apixaban, dabigatran, edoxaban, and rivaroxaban) (32). Up-to-date findings suggest that the main factors contributing to the variability in plasma DOAC concentrations are drug interactions and PGx, but scientific knowledge is still limited. The absorption of the prodrug dabigatran etexilate depends on the function of intestinal membrane protein P-glycoprotein (gene *ABCB1/MDR1*), while its conversion to the active drug form depends on hepatic carboxylesterase 1 (CES1). Interindividual variation of dabigatran plasma concentrations has been found to be affected by gene variants of the *ABCB1* rs1045642, rs4148738, and *CES1* rs2244613, but the results did not reach significance (33-35).

A significant association was revealed between rivaroxaban concentrations and CYP3A enzyme activity, with a possible role of variability in two drug transporters, ABCB1 and ABCG2 (36-38). For apixaban, the pharmacogenes of interest are *CYP3A4, ABCB1, ABCG2,* and *SULT* (39,40), while those for edoxaban are *CYP3A4, CES1, SLCO1B1,* and *ABCB1* (41,42). The clinical relevance of these and newly discovered genes should be examined in future studies focusing on drug-drug-gene interactions, especially in the elderly with comorbidities and polytherapy.

### Adenosine diphosphate (ADP) receptor antagonists – P2Y12 inhibitors

P2Y12 inhibitors clopidogrel and prasugrel are prodrugs that are metabolized by multiple CYP enzymes to their pharmacologically active metabolites, which irreversibly inhibit the ADP P2Y12 receptor (43). CYP2C19 is the main enzyme involved in clopidogrel conversion to its active metabolite. Consequently, the carriers of inactivating alleles CYP2C19*2 or *3 experience a reduced therapeutic efficacy (44,45) and higher risk of recurrent cardiovascular events. The CPIC issued dosing guideline for clopidogrel (46) (Table 1).

Interesting data have been obtained for the increased function of CYP2C19*17 (rs12248560) allele, which increases *CYP2C19* expression (heterozygote carriers are considered rapid metabolizers and homozygote carriers ultrarapid metabolizers). *17 is present in ≈ 30% of Caucasians (24,46), and the carriers treated with clopidogrel have increased active metabolite formation and therefore increased platelet aggregation inhibition and lower major adverse cardiac event (MACE) risk. Some studies also observed a higher bleeding risk (44,47-49). Due to inconsistent results, the clinical implications for *17 carriers remain uncertain and need further research. Other genetic variants that can serve as biomarkers in the individualization of clopidogrel treatment are the transporter gene *ABCB1* c.*3435C>T* (decreased clopidogrel absorption) and *CES1,* carboxylesterase-1 (increased active metabolite formation), but testing for these variants is not currently recommended (46). As PPIs are also metabolized by CYP2C19, and some of them are inhibitors of this enzyme, there is a potential for drug-drug-gene interactions (50).

There are no recommendations for genotyping in the case of other antithrombotic drugs, although some pharmacogenetics evidence connects CYPs gene variants with drug concentrations. Prasugrel is activated predominantly by CYP3A4 and CYP2B6 and in smaller degree by CYP2C19. Ticagrelor’s metabolite (AR-C124910XX), with equipotent antiplatelet effects, is a result of CYP3A4 enzymatic activity. In the PLATO and TRITON-TIMI 38 randomized studies, ticagrelor and prasugrel, respectively, showed better MACE risk reduction than clopidogrel in acute coronary syndrome (ACS) patients (51,52). However, both drugs had higher bleeding rates and higher cost and discontinuation rates (53). In clinical practice, clopidogrel is the most often used P2Y12 inhibitor. In a recent study, CYP3A4*22 low activity allele carriers had the area under the plasma concentration-time curve of ticagrelor 89% (*P* = 0.004), higher than CYP3A4*1 normal activity allele carriers (54). This markedly impaired elimination resulted in its enhanced antiplatelet effect. However, there is a lack of data on the association of *22 allele with bleeding. The impact of prasugrel and ticagrelor pharmacogenes variants, including CYP3A4*22 (rs35599367) and *SLCO1B1* (rs4149056), on clinical and safety outcomes is still unclear and warrants further research.

Our analysis of eight cases from VigiBase (World Health Organization's global Individual Case Safety Report database), describing drug-drug interactions (DDI) between rosuvastatin and ticagrelor that lead to rhabdomyolysis, pointed to several potential aspects that may result in the onset of rhabdomyolysis: old age, very high dose of rosuvastatin, DDI at the level of drug metabolic enzymes (CYPs and UGTs) and drug transporters (ABCB1, ABCG2, OATP1B1) in addition to pharmacogenetic susceptibility (55). Pharmacogenetic analysis indicated the presence of inactivating alleles: CYP3A4 *1/*22, CYP2C9 *1/*3, CYP2D6 *1/*4, UGT1A1 *28/*28, *UGT2B7 -161C/T*, *ABCB1 3435C/T,* and *ABCB1 1237C/T,* which may possibly enhance the DDI, not merely concerning rosuvastatin and ticagrelor, but also concerning other concomitant drugs, such as amiodarone and proton pump inhibitors (PPI). A recent review focused on possible mechanisms of interactions of the most widely used statins with ticagrelor, including CYP3A4 isoenzyme, glucuronidation, organic anion transporter polypeptides (OATPs), and P-glycoprotein. Although the concomitant use of statins with ticagrelor, at usually prescribed dosages, was found to be quite harmless, thoughtful approach should be applied, especially in older patients (56).

The main challenge in the management of coronary heart disease (CHD) patients with concurrent venous thromboembolism is that simultaneous application of various antithrombotic treatments could increase the bleeding risk (57). A PGx approach can help optimize antithrombotic therapy, achieve the most beneficial effects, and minimize the risk of bleeding when combining anticoagulants, antiplatelet medicines, triple antithrombotic therapy regimen, and thrombolytic drugs and treatment over longer periods.

### β-hydroxy β-methylglutaryl-CoA reductase inhibitors (statins)

Statins are used as hypolipidemics for primary and secondary prevention of cardiovascular (CV) disease and belong among the most highly prescribed drugs worldwide (58). They are commonly well tolerated, but in some patients can cause ADRs. Statins are mostly discontinued due to musculoskeletal side effects (MSE), followed by hepatic toxicity. MSE range from common myalgias ( ~ 5% patients) to increasingly severe myopathies accompanied by raised plasma creatine kinase levels ( ~ 0.1% patients), and to rare rhabdomyolysis (0.1-8.4/100 000 patient-years) (59). The occurrence of cognitive impairment or new onset of diabetes is rare and questionable, without clear clinical evidence (60). In high CV risk patients, a high- statin dose is recommended, but very often these patients are underdosed (61). As most ADRs of statins are infrequent and not serious, statins are often used continuously during a longer time period and thereby are susceptible to DDIs, which can increase the risk of statin-associated MSE and hepatic toxicity. Possible origins of statin DDIs are the use of concomitant drugs and pharmacogenetic predisposition. Therefore, the safety of statins use has been intensively studied, mainly in patients on polytherapy at risk of DDIs (62). The patients of special concern are those who cannot tolerate statins or who do not reach their low density lipoprotein cholesterol goal, since they need an additional non-statin lipid-modifying agent, such as ezetimibe or a PCSK9 inhibitor, increasing the risk of ADRs (58).

Currently, pharmacogenetic guidelines are published only for simvastatin and atorvastatin and *SLCO1B1* gene (coding for organic anion transporting polypeptides OATP1B1) (Table 1) (63). Higher statin exposure (especially with simvastatin) with greater MSE risk has been found in the carriers of *SLCO1B1* variants associated with OATP1B1 deficiency. For example, homozygous carriers of low activity allele SLCO1B1*5/*5 treated with 80 mg/d of simvastatin bear a 17-fold higher MSE risk, while heterozygous carriers (SLCO1B1*1/*5) bear a 3- to 5-fold higher risk compared with the carriers of the wild type allele (64). Since the MSE risk is multifactorial, pharmacogenetic examination has a limited positive predictive value. Pharmacogenetic tests before starting statin therapy are not recommended; however, *SLCO1B1* genotyping may be indicated if MSE signs and symptoms occur in a patient on statins. The CPIC has issued dosing guidelines (63,65). The leading risk factors for statins ADRs are genetic factors (eg, *SLCO1B1, CYP3A4/5,* and *ABCG2*), comorbidities (kidney failure, liver dysfunction, hypothyroidism, diabetes mellitus), very young or very old age, female sex, and drug-associated factors (dosage, statin type, and concomitant therapy with OATP1B1, ABCG2, and/or CYP3A4/5 inhibitors, eg, ketoconazole, cyclosporine) (59,66-68). *In vitro* studies showed that statin lactones were more potent and myotoxic than statin acids.

Statin therapy risk prediction can be considerably improved by a multifactorial approach that includes multiple genes testing along with the prediction of DDIs and other risk factors. Although pharmacogenomic analysis is progressively applied with the aim of drug therapy optimization, its real potential in elderly population on polypharmacy has not been studied so far.

A study conducted in collaboration with the Croatian Agency for Medicinal Products and Medical Devices, which recruited patients with ADRs, pointed to several genes that presented the risk of statin ADRs (69). Besides the well-known association with SLCO1B1*5 variant, results showed a significant impact of another drug transporter gene variant, *ABCG2 421C>A,* on the development of atorvastatin ADRs, which was prominent in older age patients (66). In another study investigating fluvastatin ADRs risk in renal transplant patients, we also confirmed the relevance of *ABCG2 421C>A,* along with the polymorphisms of *CYP2C9* (fluvastatin main metabolic pathway) and concomitant therapy with CYP2C9 inhibitors (70).

A recent study found multiple factors associated with atorvastatin and its metabolite concentrations, including smoking and drug-drug-gene interactions involving proton PPIs and loop diuretics, principally furosemide (71). An association between PPIs and the *CYP2C19* genotype with an impact on atorvastatin concentrations was also detected.

Our previously published case report described a similar finding (72). This case illustrated the clinical relevance of the relationship between pharmacogenetics (low activity transporter OATP1B1 and low activity enzyme CYP2C19) and DDI between atorvastatin and a PPI (pantoprazole) in the development of rhabdomyolysis with acute renal failure.

Another example from our routine genetic testing is that of a patient with polypharmacy with statins (atorvastatin followed by rosuvastatin) and ezetimibe who developed signs of hepatotoxicity (significantly elevated transaminases ALT, AST), also suggesting the relevance of pharmacogenetics interacting with DDIs (73). The patient was homozygous for *ABCG2 421A* low activity allele, predisposing for low transporter activity and impaired elimination of statins and ezetimibe into the bile. The fact that all three drugs are substrates of ABCG2 (in addition, ezetimibe is also an ABCG2 inhibitor), and genetically conditioned poor activity of ABCG2, resulted in slower drug elimination into the bile and enhanced adverse effects on the liver. More and more emerging data are pointing to *ABCG2* as a promising pharmacogenetic biomarker, not only for statins but also other drugs, predisposing for drug-drug-gene interactions (74).

In liver microsomes (animal/rat model), interactions between ticagrelor or prasugrel with statins and their impact on safety and effectiveness confirmed that the co-administration of P2Y12 inhibitors with simvastatin might significantly inhibit the CYP3A4 activity (75). The data also suggested that ticagrelor inhibited CYP3A4 activity. Since almost half of the prescribed drugs are CYP3A4 substrates, this finding could be relevant for clinical practice, especially for the treatment of older people on polypharmacy.

Regarding other cardiovascular drugs, guidelines have also been issued for metoprolol, propafenone, and flecainide, based on *CYP2D6* genotype (Table 1).

Although many antihypertensives and beta-blockers are substrates of CYP polymorphic enzymes, predominantly CYP2D6 and CYP3A4, there are no genotype-dosage recommendations. Here as well, special care should be taken when administering multiple drugs that are substrates of these enzymes, which is again especially emphasized in the elderly population. Therefore, genotyping can help in predicting the intensity of drug interactions and detect slow metabolizers, who have a significantly increased ADRs development.

## Rare gene variants

Besides common genetic polymorphisms, recent projects based on advanced sequencing methods revealed many rare genetic variants in ADME and drug response relevant genes (76). Specific pharmacogenes panels, which provide information for multiple polymorphisms including rare variants, have been developed for clinical testing and will improve pharmacogenetic analysis and ADRs prediction.

## The cost-effectiveness of pharmacogenomics approach

In the recent decade, several hundred studies have assessed the cost-effectiveness of PGx testing. Their major disadvantage was that they were undertaken after the choice of a treatment protocol, meaning that reactive genotyping was performed instead of pre-emptive testing. Furthermore, categories in economic models were not uniform and results were not comparable.

The majority of studies on CV drugs were focused on warfarin and clopidogrel, and only a smaller number investigated statins and ACE inhibitors. A recently published comprehensive systematic review by Zhu et al showed that 67% of the included high-quality studies found that CVD treatment PGx testing was cost-effective (79). However, 20% of studies observed questionable cost-effectiveness of PGx vs standard treatment, while 13% of studies were inconclusive. Zhu et al found that PGx-guided clopidogrel treatment showed cost-effectiveness in 81% of studies, and warfarin treatment in 56% (79). The data were specifically supportive in patients with ACS and AF. These findings are in agreement with the results of our study in AF patients (warfarin and *CYP2C9*/*VKORC1* genotyping) who developed ischemic stroke (31).

A newly published study (80) found that PGx (multi-gene genotyping of CYP2C9, CYP2C19, VKORC1, and SLCO1B1) was cost-effective compared with standard treatment and single gene genotyping in ACS patients with percutaneous coronary intervention (PCI) (80).

Another systematic review reported that the majority of PGx-guided treatment economic assessments were cost-effective, with an average Quality of Health Economic Studies score of 76 (score range from 0-100; >75 is high). However the review included not only CV drugs, but other drugs as well (81). In conclusion, PGx-guided CVD treatment cost-effectiveness has not yet been clearly stated.

## Conclusions

Pharmacogenomics data may improve the selection of a particular drug treatment and allow the tailoring of dose and dosing schedule to the patient's genetic profile. This can enhance drug effectiveness and reduce toxicity and thus be of enormous importance for elderly patients with comorbidities and polypharmacy.

In the field of CV diseases treatment, the progress of pharmacogenetics/PGx has increased our understanding of the molecular mechanisms involved in the toxicity and efficacy of commonly used CV drugs. Pharmacogenetics analysis and recommendations issued by several consortia enable clinical applications that can improve the prediction of VKA and clopidogrel resistance and hemorrhagic or MSE risk. This PGx approach has had a significant impact on health care efficiency and economic status of society. However, we still need to address many clinical challenges. Other tests, potentially for direct oral anticoagulants, beta-blockers, or antihypertensives can be expected. Since the majority of pharmacogenetics recommendations are based on the estimation of single drug-gene interactions, for older people with polypharmacy it is imperative that we develop methods and tools for the prediction of multiple drug-drug-gene interactions. Pharmacogenetics analysis is more accessible but is still insufficiently used in clinical medicine. Its wider implementation requires physicians’ and scientists’ knowledge and expertise concerning genetics, risk prediction, and genetic counselling. In addition, improved patients’ satisfaction with pharmacotherapy will result in better compliance and overall treatment success. We believe that in the near future, the economic evaluation of pre-emptive testing, which will include genetic panel, namely all important CV drug-gene pairs, might demonstrate its usefulness and cost-effectiveness. Specifically, data for elderly population on long term polytherapy of chronic CVD could be even more significant and favor the cost-effective PGx guided CVD treatment.

In conclusion, personalized medicine based on PGx has a potential to yield significant health and economic benefits for patients, especially the elderly, health care professionals, and society.
